# Association of triglyceride to high-density lipoprotein cholesterol and low-density lipoprotein to high-density lipoprotein cholesterol ratios with glycemic control in type 2 diabetes mellitus: A cross-sectional study from a Libyan clinical cohort

**DOI:** 10.1097/MD.0000000000049337

**Published:** 2026-06-19

**Authors:** Sahar H. Mahmoud, Fatma R. Elfargani, Nagwa Mohamed, Yousef Khader

**Affiliations:** a Department of Physiology, Faculty of Medicine, Benghazi University, Benghazi, Libya; b Department of Public Health, Faculty of Medicine, Jordan University of Science and Technology, Irbid, Jordan.

**Keywords:** dyslipidemia, glycemic control, HDL-C, inflammation, LDL-C/HDL-C ratio, TG/HDL-C ratio, triglycerides, type 2 diabetes mellitus

## Abstract

Poor glycemic control in type 2 diabetes mellitus (T2DM) is frequently associated with adverse lipid profiles, including elevated triglycerides (TG) and low high-density lipoprotein cholesterol (HDL-C). Lipid ratios, including TG/HDL-C (triglycerides/high-density lipoprotein cholesterol) and LDL-C/HDL-C (low-density/ high-density lipoprotein cholesterol), may serve as simple markers of insulin resistance and cardiometabolic risk. This study aimed to investigate the association between glycemic control and lipid parameters, including TG, HDL-C, and lipid ratios, in a Libyan cohort of T2DM patients. In this retrospective cross-sectional study, 363 T2DM patients (140 males, 223 females) aged 18 to 80 years attending a diabetic polyclinic in Benghazi between January 2022 and January 2024 were included. Clinical and biochemical data, including fasting lipid profiles and glycated hemoglobin (HbA1c), were extracted from patient records. Participants were classified according to glycemic control (HbA1c < 7% vs ≥7%). Associations between lipid parameters, lipid ratios, and glycemic control were assessed using multivariable logistic regression adjusted for age, sex, BMI, duration of diabetes, and statin use. Patients with uncontrolled HbA1c (≥7%) had significantly higher TG/HDL-C ratios (3.79 ± 2.52 vs 2.92 ± 2.42; *P* = .007) and LDL-C/HDL-C ratios (2.55 ± 1.30 vs 1.95 ± 1.05; *P* < .001) compared with those with controlled HbA1c. In multivariable logistic regression analysis, higher low-density lipoprotein cholesterol (LDL-C) levels were significantly associated with uncontrolled HbA1c (OR = 1.02; 95% CI: 1.01–1.03; *P* < .001). When lipid ratios were evaluated, the LDL-C/HDL-C ratio remained significantly associated with uncontrolled HbA1c (OR = 1.05; 95% CI: 1.02–1.09; *P* = .003). Lipid ratios, particularly LDL-C/HDL-C, were associated with poor glycemic control in type 2 diabetes mellitus; however, their clinical utility requires further validation in prospective studies. Integrated management of dyslipidemia and glycemic control is essential to reduce cardiovascular complications in this population.

## 1. Introduction

Type 2 diabetes mellitus (T2DM) is a chronic metabolic disorder characterized by hyperglycemia resulting from insulin resistance and impaired pancreatic β-cell function. T2DM is frequently accompanied by dyslipidemia, which substantially contributes to the elevated cardiovascular disease risk observed in these patients.^[1,2]^ The characteristic diabetic dyslipidemia profile consists of elevated triglycerides (TG), reduced high-density lipoprotein cholesterol (HDL-C), and increased small dense low-density lipoprotein (sdLDL) particles. These lipid abnormalities are closely associated with insulin resistance, chronic hyperglycemia, and low-grade systemic inflammation, creating a pro-atherogenic milieu.^[3,4]^

Beyond individual lipid measures, lipid ratios such as TG/HDL-C and LDL-C/HDL-C have emerged as practical markers for insulin resistance and cardiometabolic risk. The TG/HDL-C ratio correlates strongly with insulin resistance, metabolic syndrome, and poor glycemic control (HbA1c), demonstrating higher predictive value than isolated TG or HDL-C levels.^[5,6]^ Similarly, the LDL-C/HDL-C ratio provides an integrated measure of atherogenic versus protective lipoproteins and has been associated with cardiovascular risk and glycemic dysregulation in diverse populations.^[7]^ These lipid ratios are low-cost, accessible, and clinically useful tools for identifying patients at increased risk of metabolic dysfunction, particularly in resource-limited settings.

Several studies have demonstrated that the triglyceride-to-high-density lipoprotein cholesterol (TG/HDL-C) ratio is associated with poor glycemic control in individuals with type 2 diabetes mellitus. Higher TG/HDL-C ratios have been linked to elevated HbA1c levels and impaired glucose regulation. Similarly, the low-density lipoprotein cholesterol to high-density lipoprotein cholesterol (LDL-C/HDL-C) ratio has been associated with glycemic dysregulation, reflecting the balance between atherogenic and protective lipoproteins. As TG, low-density lipoprotein cholesterol (LDL-C), and HDL-C are routinely measured in clinical practice, these lipid ratios represent simple, inexpensive, and accessible markers for assessing metabolic abnormalities in patients with T2DM.^[6,8,9]^

Management of dyslipidemia in T2DM often includes pharmacologic therapy with statins, which are widely recommended to reduce cardiovascular risk. However, evidence from meta-analyses indicates that statin therapy may be associated with a modest increase in HbA1c and measures of insulin resistance in people with diabetes, highlighting the need to consider potential effects on glycemic control when interpreting lipid–glycemia relationships in clinical studies.^[10,11]^

Despite extensive global evidence, data on the relationship between lipid ratios and glycemic control in North African populations, including Libya, remain scarce. A study conducted at the Al-Khums Diabetic Center in Al-Khums, Libya, found that over 60% of patients with T2DM had dyslipidemia, with low HDL-C and elevated triglycerides frequently observed alongside poor glycemic control.^[12]^ Separately, research in Derna, Libya, reported that more than two-thirds of patients with T2DM exhibited poor glycemic control and significant lipid abnormalities, highlighting the clinical relevance of monitoring lipid profiles in Libyan diabetic populations.^[13]^ These findings underscore the importance of investigating the interplay between lipid ratios and glycemic control in local cohorts to guide risk stratification and the development of therapeutic interventions tailored to the Libyan population.

Therefore, the present study aimed to determine the association between glycemic control and lipid parameters, including TG, HDL-C, TG/HDL-C ratio, and LDL-C/HDL-C ratio, in a cohort of patients with T2DM attending a diabetic clinic in Benghazi, Libya. By examining these relationships, we assessed the association between lipid ratios and glycemic control, providing insight into their potential relevance in the clinical evaluation of patients with T2DM. We hypothesized that higher TG/HDL-C and LDL-C/HDL-C ratios would be associated with poor glycemic control in this cohort.

## 2. Methods

### 2.1. Study design and participants

This retrospective cross-sectional study was conducted at the National Center for Diagnosis and Treatment of Diabetes in Benghazi, Libya, between January 2022 and January 2024. A total of 363 patients diagnosed with type 2 diabetes mellitus were included in the study, of whom 140 were males and 223 were females. Eligible participants were adults aged between 18 and 80 years with available clinical and laboratory records. Type 2 diabetes mellitus was diagnosed according to the American Diabetes Association criteria, based on fasting plasma glucose ≥ 126 mg/dL, and/or glycated hemoglobin ≥ 6.5%, and/or use of antidiabetic medications.^[14]^

Patients were excluded if they had acute or chronic infections within 2 weeks before testing, autoimmune or hematologic disorders, malignancy, pregnancy, or severe hepatic or renal impairment. Severe renal impairment was defined as an estimated glomerular filtration rate (eGFR) < 30 mL/min/1.73 m^2^, corresponding to stage 4 to 5 chronic kidney disease according to the Kidney Disease: Improving Global Outcomes (KDIGO) classification.^[15]^

### 2.2. Data collection

Patient medical records were used to gather demographic and clinical information, including age, sex, body mass index (BMI), duration of diabetes, and medication history. BMI was calculated as weight in kilograms divided by height in meters squared (kg/m^2^) and classified according to WHO guidelines: normal (18.5–24.9 kg/m^2^), overweight (25–29.9 kg/m^2^), and obese (≥30 kg/m^2^). Medication history included statin therapy; 151 of 363 patients (41.6%) were on Simvastatin (10–40 mg/day), Atorvastatin (10–40 mg/day), or Rosuvastatin (10–40 mg/day).

Laboratory measurements included fasting plasma glucose, HbA1c, hemoglobin, and lipid profile parameters, including total cholesterol, low-density lipoprotein cholesterol (LDL-C), high-density lipoprotein cholesterol (HDL-C), and triglycerides (TG). All laboratory measurements were performed in the central laboratory of the National Center for Diagnosis and Treatment of Diabetes using standardized automated methods according to the manufacturer’s protocols. Laboratory quality control procedures were routinely applied to ensure the accuracy and reliability of results.

### 2.3. Definitions

The TG/HDL-C ratio was determined by dividing triglyceride levels by HDL-C levels, while the LDL-C/HDL-C ratio was calculated by dividing LDL-C levels by HDL-C levels. Lipid parameters were reported in milligrams per deciliter (mg/dL). Participants were categorized according to glycemic control based on their HbA1c levels. Individuals with HbA1c values below 7% were classified as having controlled diabetes, whereas those with HbA1c levels of 7% or higher were considered to have uncontrolled diabetes.

### 2.4. Ethical considerations

This study was approved by the Training and Development National Center for Diagnosis and Treatment of Diabetes in the Eastern Region (Approval Number: 2024-30) and conducted according to the ethical standards outlined in the Helsinki Declaration of 1964.

### 2.5. Statistical analysis

Continuous variables were described using mean ± standard deviation, whereas categorical variables were expressed as numbers and percentages. Comparisons between patients with controlled and uncontrolled HbA1c were performed using independent sample t-tests for continuous variables and chi-square tests for categorical variables.

Multivariable logistic regression analyses were conducted to identify factors associated with uncontrolled HbA1c. Two models were constructed. The first model included LDL-C and HDL-C as separate variables, while the second model included the LDL-C/HDL-C ratio as a combined lipid indicator. Both models were adjusted for potential confounding factors, including age, sex, body mass index category, duration of diabetes, and statin use.

Adjusted odds ratios along with their 95% confidence intervals were reported. Model performance was assessed using Nagelkerke R^2^ and overall classification accuracy. A *P*-value < .05 was considered statistically significant. The data were analyzed using SPSS version 24.

## 3. Results

### 3.1. Participant characteristics by HbA1c control status

A total of 363 patients with type 2 diabetes were included in the analysis (140 males and 223 females), of whom 75 (20.7%) had controlled HbA1c (<7%) and 288 (79.3%) had uncontrolled HbA1c (≥7%).

Table 1 compares the clinical and biochemical characteristics of participants according to HbA1c control status. The mean age of participants was similar between the 2 groups (51 ± 14 years among controlled vs 50 ± 12 years among uncontrolled; *P* = .613). Similarly, BMI and duration of diabetes did not differ significantly between the groups. However, several metabolic parameters differed significantly between the 2 groups. Participants with uncontrolled HbA1c had higher total cholesterol (175 ± 46 vs 156 ± 33 mg/dL; *P* = .001), higher LDL cholesterol (106 ± 39 vs 85 ± 28 mg/dL; *P* < .001), and higher triglycerides (154 ± 91 vs 124 ± 74 mg/dL; *P* = .008). Lipid ratios were also significantly higher in the uncontrolled group, including the TG/HDL-C ratio (3.8 ± 2.5 vs 2.9 ± 2.4; *P* = .007) and the LDL-C/HDL-C ratio (2.6 ± 1.3 vs 2.0 ± 1.1; *P* < .001). HDL-C levels did not differ significantly between groups (*P* = .079).

**Table 1:** Comparison of clinical and biochemical characteristics by HbA1c control status.

Variable	Controlled (HbA1c < 7%) Mean ± SD N = 75	Uncontrolled (HbA1c ≥ 7%) Mean ± SD N = 288	*P*-value
Age (yr)	51 ± 14	50 ± 12	.613
BMI (kg/m^2^)	33 ± 7	32 ± 8	.197
Duration of DM (yr)	7 ± 7	6 ± 7	.826
FPG (mg/dL)	118 ± 30	165 ± 65	<.001
HbA1c (%)	6.3 ± 0.6	9.5 ± 4.1	<.001
Hemoglobin (g/dL)	12.8 ± 1.8	13.4 ± 1.8	.010
Total cholesterol (mg/dL)	156 ± 33	175 ± 46	.001
HDL-C (mg/dL)	48 ± 16	45 ± 13	.079
LDL-C (mg/dL)	85 ± 28	107 ± 39	<.001
Triglycerides (mg/dL)	124 ± 74	154 ± 91	.008
TG/HDL-C ratio	2.92 ± 2.42	3.79 ± 2.52	.007
LDL-C/HDL-C ratio	1.95 ± 1.05	2.55 ± 1.30	<.001

BMI = body mass index, DM = diabetes mellitus, FPG = fasting plasma glucose, HbA1c = glycated hemoglobin, HDL-C = high-density lipoprotein cholesterol, LDL-C = low-density lipoprotein cholesterol, SD = standard deviation, TG = triglycerides.

### 3.2. Association between categorical variables and HbA1c control

The association between selected categorical variables and HbA1c control status is shown in Table 2. No significant differences were observed between controlled and uncontrolled groups with respect to sex (*P* = .258), statin use (*P* = .207), age category (*P* = .151), BMI category (*P* = .118), or duration of diabetes (*P* = .324). About 151 out of 363 patients were on statin therapy, such as Simvastatin, Atorvastatin, and Rosuvastatin.

**Table 2:** Association between participant characteristics and HbA1c control status.

Variable	Controlled (HbA1c < 7%) n (%)	Uncontrolled (HbA1c ≥ 7%) n (%)	Total n (%)	*P*-value
Sex				
Female	50 (66.7)	169 (59.5)	219 (61.0)	.258
Male	25 (33.3)	115 (40.5)	140 (39.0)	
Statin use				
No	39 (52.0)	173 (60.1)	212 (58.4)	.207
Yes	36 (48.0)	115 (39.9)	151 (41.6)	
Age				
<50 yr	30 (40.0)	142 (49.3)	172 (47.4)	.151
≥50 yr	45 (60.0)	146 (50.7)	191 (52.6)	
BMI				
Normal	5 (6.7)	45 (15.6)	50 (13.8)	.118
Overweight	20 (26.7)	77 (26.7)	97 (26.7)	
Obese	50 (66.7)	166 (57.6)	216 (59.5)	
Duration of diabetes				
Newly diagnosed (<2 yr)	18 (24.3)	95 (33.2)	113 (31.4)	.324
2–5 yr	25 (33.8)	81 (28.3)	106 (29.4)	
>5 yr	31 (41.9)	110 (38.5)	141 (39.2)	

BMI = body mass index; HbA1c = glycated hemoglobin.

### 3.3. Multivariable logistic regression analysis

Two multivariable logistic regression models were constructed to identify factors associated with uncontrolled HbA1c. In the first model (Table 3), which included LDL-C and HDL-C as separate variables, higher LDL-C levels were significantly associated with uncontrolled HbA1c (OR = 1.02; 95% CI: 1.01–1.03; *P* < .001). Additionally, individuals who were overweight had higher odds of uncontrolled HbA1c compared with obese participants (OR = 3.49; 95% CI: 1.12–10.82; *P* = .031). Other variables, including sex, statin use, age category, duration of diabetes, HDL-C, and triglycerides, were not significantly associated with HbA1c control. This model showed moderate explanatory power (Nagelkerke *R*^2^ = 0.159) with an overall classification accuracy of 80.3%.

**Table 3:** Multivariable logistic regression analysis of factors associated with uncontrolled HbA1c (≥7%).

Variable	Adjusted OR	95% CI	*P*-value
Sex (Female vs Male)	0.91	0.47–1.76	.778
Statin use (Yes vs No)	1.36	0.76–2.44	.301
Age category (≤50 vs ≥50 yr)	1.20	0.68–2.12	.538
BMI category			
Obesity	1.00		
Overweight	3.49	1.12–10.82	.031
Normal	1.21	0.64–2.28	.552
Duration of diabetes			
Newly diagnosed	1.00		
2–5 yr	0.74	0.36–1.52	0.409
>5 yr	1.16	0.56–2.39	0.682
HDL-C (mg/dL)	0.99	0.97–1.01	.323
LDL-C (mg/dL)	1.02	1.01–1.03	<.001
Triglycerides (mg/dL)	1.00	1.00–1.01	.100

BMI = body mass index, CI = confidence interval, HDL-C = high-density lipoprotein cholesterol, LDL-C = low-density lipoprotein cholesterol, OR = odds ratio.

In the second model (Table 4), the LDL-C/HDL-C ratio was used instead of LDL-C and HDL-C individually. The LDL-C/HDL-C ratio remained significantly associated with uncontrolled HbA1c. For every 0.1-unit increase in the LDL-C/HDL-C ratio, the odds of having uncontrolled HbA1c increased by 5% (OR = 1.05; 95% CI: 1.02–1.09; *P* = .003). Overweight participants again had higher odds of uncontrolled HbA1c compared with obese participants (OR = 3.69; 95% CI: 1.20–11.31; *P* = .022). No significant associations were observed for sex, statin use, age category, duration of diabetes, or triglyceride levels. This model demonstrated slightly lower explanatory power (Nagelkerke *R*^2^ = 0.128) and classification accuracy (78.9%).

**Table 4:** Multivariable logistic regression model for predictors of uncontrolled HbA1c (≥7%) using LDL-C/HDL-C ratio.

Variable	Adjusted OR	95% CI	*P*-value
Sex (Female vs Male)	1.06	0.57–1.97	.855
Statin use (Yes vs No)	1.29	0.73–2.29	.386
Age category (≤50 vs ≥50 yr)	1.21	0.69–2.14	.499
BMI category			
Obesity	1.00		
Overweight	3.69	1.20–11.31	.022
Normal	1.21	0.65–2.25	.539
Duration of diabetes			
Newly diagnosed	1.00		
2–5 yr	0.73	0.36–1.50	.397
>5 yr	1.02	0.51–2.04	.959
Triglycerides (mg/dL)	1.00	1.00–1.01	.092
LDL-C/HDL-C ratio	1.05*	1.02–1.09	.003

BMI = body mass index, CI = confidence interval, HDL-C = high-density lipoprotein cholesterol, LDL-C = low-density lipoprotein cholesterol, OR = odds ratio.

* OR represents the change in odds associated with a 0.1-unit increase in the LDL-C/HDL-C ratio.

## 4. Discussion

This study demonstrated that higher LDL-C levels were associated with uncontrolled HbA1c, and the LDL-C/HDL-C ratio was also associated with poor glycemic control. In contrast, triglyceride and HDL-C levels considered individually were not associated with HbA1c status.

In our cohort, total cholesterol was higher in patients with uncontrolled HbA1c compared with those with controlled glycemia. This observation aligns with previous studies in T2DM populations, which have reported associations between total cholesterol levels and poor glycemic control, suggesting that hyperglycemia often coexists with a more atherogenic lipid profile.^[16,17]^

Our findings are consistent with studies reporting associations between the LDL-C/HDL-C ratio and glycemic status in individuals with T2DM. For example, Artha and colleagues reported that the LDL-C/HDL-C ratio identified individuals with uncontrolled HbA1c with reasonable sensitivity and specificity.^[18]^ Similarly, another cross-sectional study showed that as HbA1c levels increased, both LDL-C and the LDL-C/HDL-C ratio rose in parallel, whereas HDL-C alone showed a weaker correlation with glycemic status.^[9]^ These observations suggest that the LDL-C/HDL-C ratio reflects aspects of diabetic dyslipidemia that may parallel glycemic status.

Several studies have shown positive relationships between the LDL-C/HDL-C ratio and HbA1c in T2DM patients, even after adjustment for confounders such as age, BMI, and total cholesterol.^[18]^ A Middle Eastern cohort similarly demonstrated rising LDL-C/HDL-C ratios with increasing HbA1c.^[9]^ In addition, LDL-C/HDL-C has been associated with markers of insulin resistance, such as HOMA-IR.^[19]^ While these studies support a relationship between lipid ratios and glycemic measures, the strength and consistency of this association vary across populations and study designs, likely reflecting differences in demographics, treatment patterns, and metabolic characteristics.

Mechanisms linking dyslipidemia and hyperglycemia have been proposed in the literature, including pathways involving oxidative stress, endothelial dysfunction, and β-cell impairment.^[20,21]^ However, these mechanisms were not evaluated in the present study, as no biomarkers of inflammation, insulin resistance, or endothelial function were measured. Therefore, these explanations should be considered literature-based hypotheses rather than interpretations derived from this dataset.

Although the TG/HDL-C ratio is frequently proposed as a surrogate marker of insulin resistance and cardiometabolic risk, in our cohort, it did not remain associated with HbA1c after accounting for LDL-C–related measures and other variables. In unadjusted analyses, individuals with uncontrolled HbA1c had higher TG/HDL-C ratios; however, this relationship attenuated in multivariable models, suggesting that LDL-C–centered lipid measures were more closely aligned with glycemic status in this population.

The published literature on TG/HDL-C and glycemic control shows notable variability across studies. Some cohorts have demonstrated strong associations between TG/HDL-C, HbA1c, and insulin resistance markers,^[6,22]^ whereas in broader or multi-ethnic populations, these associations weaken after adjusting for LDL-C levels, BMI, and other metabolic factors.^[23–25]^ These discrepancies likely reflect demographic differences, methodological heterogeneity, and variations in metabolic profiles across populations. Therefore, while TG/HDL-C may reflect aspects of metabolic dysfunction, its association with chronic glycemic control appears to be context-dependent rather than consistent across all settings.

The relationship between statin therapy and glycemic control remains debated. Some studies report minimal impact of statins on HbA1c,^[26]^ whereas others suggest possible associations with higher glucose levels.^[27]^ In the present study, although statin type and dose were recorded, the absence of detailed information, duration, and adherence limits the interpretation of this relationship, as variations in these factors may affect lipid levels and glycemic status.

The observation regarding BMI should also be interpreted cautiously. The wide confidence intervals suggest statistical instability, and the study design does not allow meaningful exploration of complex relationships between BMI and glycemic outcomes. Therefore, this finding should not be interpreted as evidence supporting an “obesity paradox.”^[28]^

No meaningful associations were observed for sex, age, or duration of diabetes in relation to HbA1c status. Similar findings have been reported in other clinical studies where demographic factors were less informative for glycemic stratification once metabolic variables were considered.^[29,30]^

Several factors should be considered when interpreting these findings. Data on glucose-lowering therapies were not available, and different treatment regimens could influence HbA1c independently of lipid status. Variability in statin use was not fully characterized, and lifestyle factors such as diet and physical activity were not assessed. These unmeasured variables may partly explain the observed associations. Furthermore, the retrospective cross-sectional design precludes causal inference, and data from a single clinic in Benghazi may limit generalizability.

Overall, these findings add to the body of evidence describing associations between lipid abnormalities – particularly LDL-C and the LDL-C/HDL-C ratio – and glycemic status in patients with T2DM, while underscoring the need for cautious interpretation within the methodological limitations of the study.

## 5. Conclusion

In this cross-sectional cohort of Libyan patients with type 2 diabetes mellitus, higher LDL-C levels and higher LDL-C/HDL-C ratios were observed among individuals with poorer glycemic control. These findings indicate a relationship between dyslipidemia and hyperglycemia within this population. However, due to the retrospective cross-sectional design, these associations should not be interpreted as causal or predictive. Future prospective studies incorporating comprehensive metabolic, therapeutic, and lifestyle data, as well as formal predictive analyses, are needed to determine whether lipid ratios have potential value in glycemic risk assessment.

## Acknowledgments

We sincerely thank the Training and Development National Center for Diagnosis and Treatment of Diabetes in the Eastern Region for their support in providing the data necessary for this study.

## Author contributions

**Conceptualization:** Sahar H. Mahmoud.

**Data curation:** Sahar H. Mahmoud, Yousef Khader.

**Formal analysis:** Sahar H. Mahmoud, Yousef Khader.

**Funding acquisition:** Sahar H. Mahmoud.

**Investigation:** Sahar H. Mahmoud, Nagwa Mohamed.

**Methodology:** Sahar H. Mahmoud, Fatma R. Elfargani, Yousef Khader.

**Project administration:** Sahar H. Mahmoud.

**Resources:** Sahar H. Mahmoud, Fatma R. Elfargani, Nagwa Mohamed.

**Software:** Yousef Khader.

**Supervision:** Sahar H. Mahmoud, Fatma R. Elfargani, Nagwa Mohamed, Yousef Khader.

**Validation:** Sahar H. Mahmoud, Fatma R. Elfargani, Nagwa Mohamed, Yousef Khader.

**Visualization:** Sahar H. Mahmoud.

**Writing – original draft:** Sahar H. Mahmoud.

**Writing – review & editing:** Sahar H. Mahmoud, Yousef Khader.
